# MeJA-Induced Plant Disease Resistance: A Review

**DOI:** 10.3390/plants15010169

**Published:** 2026-01-05

**Authors:** Lifeng Xiao, Yuting Li, Lingyan Cui, Jie Deng, Qiuyue Zhao, Qin Yang, Sifeng Zhao

**Affiliations:** 1 Provincial Famous Teacher Yang Qin Studio, Kaili 556000, China; xiaolifeng3905@163.com (L.X.); cuily20012005@163.com (L.C.); dengjie830@126.com (J.D.); 2 Guizhou Key Laboratory of Molecular Breeding for Characteristic Horticultural Crops, Kaili 556000, China; 3 School of Life and Health Science, Kaili University, Kaili 556011, China; lyt2338981479@163.com; 4 Key Laboratory of Oasis Agricultural Pest Management and Plant Protection Resources Utilization, Shihezi University, Shihezi 832003, China; zhaoqiuyue1@stu.shzu.edu.cn

**Keywords:** methyl jasmonate, signal transduction, induced resistance, disease management

## Abstract

This review offers a comprehensive analysis of the extensive research on methyl jasmonate (MeJA)-induced plant disease resistance. It aims to elucidate the signal transduction pathways, interactions with other phytohormones, regulation of related gene expression, and the fundamental mechanisms contributing to plant disease resistance. The review provides a detailed examination of MeJA-induced defense responses and the sustainability of the induced resistance. Furthermore, it assesses the practical applications and current status of MeJA across various plant species and explores potential research directions in disease management. It serves as a systematic reference for a deeper understanding of MeJA-induced plant disease resistance and holds significant importance for advancing further developments in the field.

## 1. Introduction

**Figure 1:**
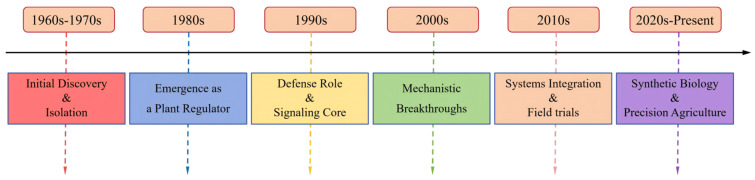
A timeline of key discoveries in JA/MeJA research.

Methyl jasmonate (MeJA) is a naturally occurring plant growth regulator extensively involved in various plant life processes, initially recognized for its contribution to the floral fragrance composition of plants such as jasmine [1]. Since the late 20th century, MeJA has been definitively identified as a crucial hormone signaling molecule within plants (Figure 1). It plays a significant role in regulating defensive responses to both biotic and abiotic stresses [2,3]. Owing to its fundamental signaling function, the exogenous application of MeJA effectively simulates stress signals, artificially activating various physiological defense pathways in plants. Consequently, over the past few decades, MeJA has been extensively utilized in both academic research and agricultural practices as a classic and highly efficient chemical inducer for investigating and enhancing the synthesis of secondary metabolites, disease resistance, and stress tolerance in plants [4,5,6].

**Figure 2:**
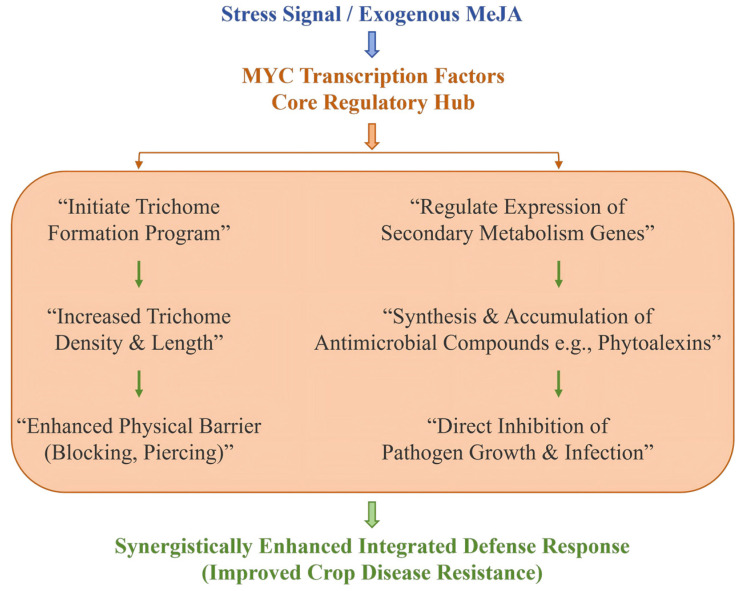
MeJA-induced crop defense via coordinating developmental and chemical pathways.

As illustrated in Figure 2, upon perception of stress or exogenous application, MeJA activates the core transcription factors of the JA signaling pathway, such as MYC2/3/4 [7,8]. These MYC factors then orchestrate a dual defense strategy. On one hand, they promote developmental defense by initiating trichome formation, thereby enhancing physical barriers against pests and pathogens [9,10]. On the other hand, they simultaneously upregulate chemical defense pathways, leading to the biosynthesis and accumulation of antimicrobial phytoalexins and other defensive metabolites [11,12,13]. This coordinated response highlights the integrative role of MeJA and its downstream transcription factors in linking morphological adaptations with metabolic reprogramming to confer enhanced disease resistance [8,14].

**Figure 3:**
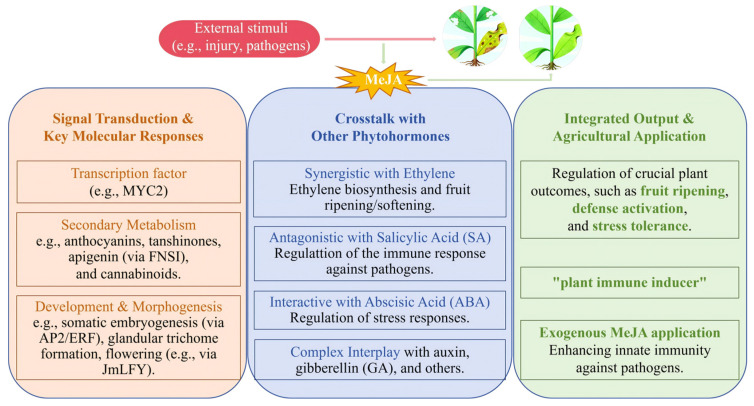
A schematic overview of the core mechanisms of Methyl Jasmonate (MeJA) [15,16,17,18,19,20,21,22,23,24,25,26,27,28,29,30].

As illustrated in Figure 3, MeJA plays a pivotal role in plant signaling networks, modulating responses to damage, growth and development, as well as numerous other physiological processes [15]. Upon exposure to external stimuli, plants activate the synthesis of MeJA. As a significant signaling compound, MeJA modulates physiological and biochemical responses by influencing gene expression and signal transduction pathways. For example, MYC2, a key transcription factor in the jasmonic acid (JA) pathway, is integral to MeJA-mediated responses, regulating plant activities by directly or indirectly influencing metabolite synthesis and aiding plants in coping with biotic and abiotic stresses through modulation of enzyme activities and gene expression levels [16]. Research has demonstrated that MeJA regulated anthocyanin biosynthesis through synergistic interactions with light signals, which elucidated its complex regulatory mechanisms in apple fruit coloration [17]. In longan (*Dimocarpus longan* Lour.), the APETALA2/ethylene response factor family is modulated by hormonal signaling molecules, including MeJA, as well as stress-responsive cis-elements, contributing to somatic embryogenesis and tissue development [18]. Flavone synthase I (FNSI), a pivotal enzyme in apigenin biosynthesis, possesses a promoter sequence enriched with MeJA-responsive elements, among others. Variability among different celery (*Apium graveolens*) varieties suggests the involvement of MeJA in related biosynthetic pathways [19]. In *Cannabis sativa*, MeJA application enhanced glandular trichome formation and modulated the accumulation of secondary metabolites through the regulation of gene expression [20]. Furthermore, a negative regulatory transcription factor associated with the MeJA signaling pathway has been identified in *Salvia miltiorrhiza* hairy roots, which played a role in regulating tanshinone biosynthesis [21]. These findings offer significant insights into the molecular mechanisms underlying MeJA signal transduction in the regulation of plant secondary metabolism.

The intricate interactions among plant hormones meticulously regulate plant growth, development, and responses to environmental stresses. MeJA engages with the signaling pathways of various endogenous hormones, thereby influencing defense mechanisms and rubber biosynthesis in rubber trees [22]. In *Brassica napus*, an increase in cyclic adenosine monophosphate (cAMP) signaling significantly impacts the levels of plant hormones, including MeJA [23]. In apple fruits, MeJA modulates the expression of the MdMYC2 gene, which in turn affects genes associated with ethylene (ET) biosynthesis and transcription factors, ultimately influencing the fruit ripening process [24]. Furthermore, MeJA treatment induces a negative feedback regulation of FAR-RED INSENSITIVE 219 (FIN219) levels in *Arabidopsis*, where FIN219 is pivotal in integrating signals from multiple hormones, suggesting that the interplay of various plant hormones governs hypocotyl and root elongation in *Arabidopsis* seedlings [25]. Under osmotic stress, *Alhagi sparsifolia* Shap adjusts the levels of plant hormones such as abscisic acid (ABA) and JA. Additionally, multiple plant hormones interact with rhizosphere microbial communities to collaboratively assist plants in coping with environmental stress [26]. The promoter sequence of the JmLFY gene in *Juglans mandshurica* is responsive to induction by various hormones, including MeJA, and its expression is modulated by multiple hormonal signals, playing a crucial role in the regulation of flowering in *Juglans mandshurica* [27]. A combined application of exogenous ET and MeJA significantly enhances ethylene synthesis in kiwifruit by activating the expression of associated genes, thereby accelerating fruit softening and ripening [28]. This suggests that MeJA can augment the role of ET in the fruit ripening process by regulating key genes within the ET biosynthesis pathway. In *Phaseolus coccineus* L. suspension-cultured cells and root tip segments, MeJA was shown to inhibit the uptake of ABA, indicating a potential interaction between MeJA and ABA transport mechanisms [29]. The interaction between MeJA and salicylic acid (SA) in plant defense responses has been substantiated, with studies demonstrating that MeJA and SA exhibit a mutually inhibitory relationship during the defense against *Colletotrichum graminicola* in maize. The presence of MeJA inhibits the activation of SA-related defense responses, thereby increasing plant susceptibility to the pathogen [30].

The exogenous application of MeJA functions as an environmentally sustainable “plant immune inducer” by pre-activating plant defense mechanisms and initiating a series of disease resistance responses, thereby effectively safeguarding plants against pathogen infections. This review systematically synthesizes the latest research advancements concerning MeJA-induced plant disease resistance, with a focus on elucidating its signal transduction pathways, interactions with other phytohormones, and the molecular mechanisms underlying induced resistance. Furthermore, it evaluates the potential applications and challenges associated with MeJA. This research holds significant importance for enhancing the understanding of plant immunity theory and provides a crucial scientific foundation and application prospects for the development of eco-friendly disease control strategies, thereby promoting sustainable agricultural practices.

## 2. Mechanism of MeJA-Induced Disease Resistance

### 2.1. Direct Inhibitory Effect of MeJA on Plant Pathogens

MeJA exerts a direct inhibitory effect on plant pathogens, impacting processes such as pathogen growth, development, and toxin production as demonstrated in several studies.

Specifically, in wheat, MeJA was shown to inhibit the growth and conidiation of *Fusarium graminearum*. The reduction in Fusarium head blight (FHB) damage by MeJA was attributed to its ability to inhibit the synthesis of the deoxynivalenol (DON) toxin, rather than enhancing the wheat’s resistance response. Treatment with MeJA has been observed to reduce infection by the wild-type *F. graminearum* in wheat coleoptiles, while exhibiting no significant effect on infection by the DON-deficient mutant strain, suggesting that MeJA significantly influences the expression of genes associated with DON biosynthesis [31]. Furthermore, MeJA treatment has been found to reduce protein kinase A (PKA) activity and intracellular cAMP levels in *F. graminearum*, indicating that MeJA may exert its antifungal effects through the cAMP-PKA signaling pathway. The observation that exogenous cAMP mitigates the inhibitory effect of MeJA on DON production further corroborates the involvement of this signaling pathway [32].

Moreover, in maize, researchers have identified a novel Bowman-Birk type protease inhibitor (BBTI) protein that is regulated by the MeJA pathway and demonstrates significant antifeedant activity against the Asian corn borer. The expression of the BBTI protein is significantly induced by both exogenous MeJA application and herbivory by the Asian corn borer. This study elucidates the synthesis and regulatory mechanisms of BBTI within the JA pathway [33].

These findings enhance the understanding of the molecular mechanisms by which MeJA regulates plant–pathogen interactions, offering new insights for the development of environmentally sustainable strategies.

### 2.2. MeJA-Induced Defense Responses in Different Plants

**Figure 4:**
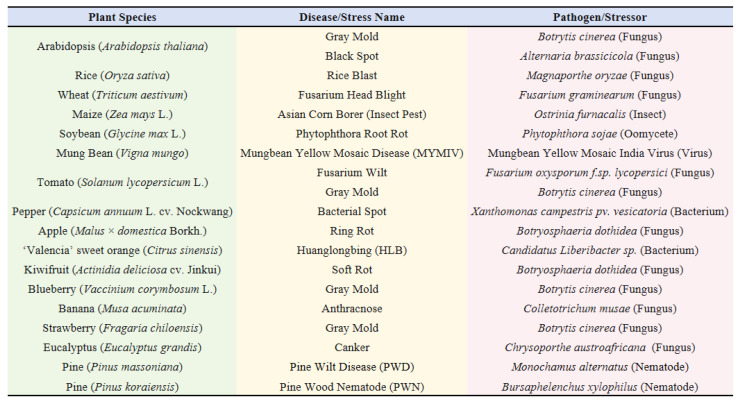
Examples of Plant Disease Resistance Induced by MeJA [34,35,36,37,38,39,40,41,42,43,44,45,46,48,49,51,55,57,58].

The MeJA signaling pathway is integral to plant responses to external stimuli and the activation of defense mechanisms, serving a pivotal function in plant immune responses against pathogens. Research indicates that upon pathogen attack, MeJA can initiate signal transduction pathways within the plant, triggering a series of complex defense responses, activating systemic defense mechanisms, and enhancing the plant’s resistance to pathogens. Collectively, MeJA acts as a powerful “plant vaccine”, and its application must be crop-specific (Figure 4).

In *Arabidopsis*, the transcript levels of a specific class of genes encoding antifungal peptides were differentially regulated following infection by the pathogen *Botrytis cinerea* or subsequent to MeJA treatment. Transgenic *Arabidopsis* plants exhibit increased resistance to infections by *B. cinerea* and *Alternaria brassicicola*, suggesting their involvement in plant defense responses, with MeJA likely playing a regulatory role in this process [34].

In rice, the overexpression of the gene OsCBSX3, has been shown to enhance resistance to the blast fungus *Magnaporthe oryzae*. Upon inoculation with *M. oryzae* and the exogenous application of SA and MeJA, OsCBSX3 transcripts were significantly upregulated. Homozygous T3 transgenic rice lines overexpressing OsCBSX3 demonstrated markedly enhanced resistance to *M. oryzae* inoculation, accompanied by increased transcript levels of immune-related marker genes, including PR1a, PR1b, and PR5, in the inoculated plants [35]. These findings suggest that OsCBSX3 functions as a positive regulator in rice resistance to *M. oryzae*, operating through the synergistic action of SA and JA-mediated signaling pathways. Furthermore, an antibiotic biosynthesis monooxygenase (Abm) in *M. oryzae* was capable of converting endogenous free JA into 12-hydroxy-JA (12OH-JA), which aided the pathogen in evading host defense responses. The loss of Abm resulted in the accumulation of MeJA, which induced host defense mechanisms, whereas the exogenous addition of 12OH-JA significantly attenuated the immune response induced by the Abm deletion in rice. This indicates that *M. oryzae* interferes with host defense by modifying JA, highlighting the importance of the interaction between MeJA and other forms of jasmonate in plant immunity [36].

In wheat, the green leaf volatile Z-3-hexenyl acetate (Z-3-HAC) has been demonstrated to enhance resistance to *Fusarium graminearum* by activating defense responses dependent on methyl jasmonate (MeJA). Research findings indicate that wheat pretreated with Z-3-HAC exhibited a 40% reduction in necrotic spikelets, significantly smaller necrotic lesions on seedling leaves, and a marked inhibition of fungal proliferation within the plant. The defense mechanism against *F. graminearum* is modulated by salicylic acid (SA) during the initial stages of infection and by JA in the later stages [37], underscoring the critical role of hormonal dynamics in influencing plant health and disease resistance.

In maize leaves, treatment with MeJA resulted in alterations in the expression of a suite of defense-related proteins, including specific expression among 62 MeJA-responsive proteins. These proteins performed a variety of biological functions, encompassing photosynthesis, energy metabolism, protein folding and degradation, stress defense, and redox responses. Feeding Asian corn borers with a fusion of four genes encoding these proteins revealed that the recombinant proteins of pathogenesis-related protein 1 (PR1) and thioredoxin M-type chloroplast precursor (TRXM) significantly impeded larval and pupal development, indicating that MeJA not only induces plant defense mechanisms against insects but also enhances the production of toxic proteins [38].

In research investigating the resistance of soybean (*Glycine max* L.) to *Phytophthora sojae*, the GmWRKY40 gene was identified and characterized. The expression of this gene was upregulated in response to *P. sojae* infection, MeJA, ET, SA, and abscisic acid. Silencing the GmWRKY40 gene in soybean hairy roots resulted in increased susceptibility to *P. sojae* infection, which was associated with a reduction in reactive oxygen species (ROS) accumulation and altered expression of certain oxidation-related genes. This suggests that GmWRKY40 functions as a positive regulator in soybean resistance to *P. sojae* by modulating hydrogen peroxide accumulation and the JA signaling pathway [39]. Similarly, in studies examining mung bean (*Vigna mungo*) resistance to *Mungbean Yellow Mosaic India Virus* (MYMIV), the exogenous application of varying concentrations of MeJA was found to reduce malondialdehyde levels, enhance membrane stability, increase the expression of antioxidant enzymes and defense marker genes, and decrease the expression of the MYMIV coat protein gene. These findings indicate that MeJA can effectively induce tolerance to MYMIV in mung bean [40].

In tomato (*Solanum lycopersicum*), research has demonstrated that the transcript of the peroxidase gene LePrx17, expressed in immature tomato fruits, was upregulated in response to JA and pathogen infection. Similarly, the expression of LePrx09 is induced by ethephon, SA, JA, among other factors. Tomato plants that overexpress LePrx09 exhibited enhanced resistance to hydrogen peroxide (H_2_O_2_) stress, suggesting a potential role for these genes in the regulation of fruit growth and disease resistance [41]. Furthermore, the treatment of tomato seeds with MeJA has been shown to enhance resistance to *Fusarium oxysporum* f. sp. *lycopersici*. This treatment significantly increases the levels of phenolic compounds, such as SA, kaempferol, and quercetin, in seedlings. It also upregulated genes such as phenylalanine ammonia-lyase (PAL5) and benzoic acid/salicylic acid carboxyl methyltransferase (BSMT), while downregulating the isochorismate synthase (ICS) gene. These findings indicate that MeJA influences the synthesis of phenolic compounds by modulating the expression of related genes, thereby enhancing resistance to fruit diseases [42]. These genetic and biochemical changes may serve as molecular markers for resistance. Additionally, low concentrations of oxalic acid (OA) secreted by *Bacillus cereus* AR156 can induce tomato resistance to gray mold through the JA/ET signaling pathway [43].

In pepper, the bZIP transcription factor encoded by the CabZIP2 gene was integral to plant defense mechanisms against bacterial pathogens. This gene was upregulated following infection by a virulent strain of *Xanthomonas campestris* pv. *vesicatoria* and is also induced by defense-related phytohormones such as SA, MeJA, and ethylene. Silencing of CabZIP2 in pepper plants results in increased susceptibility to infection by virulent strains, whereas overexpression of CabZIP2 in *Arabidopsis thaliana* enhances resistance to *Pseudomonas syringae* pv. *tomato* DC3000. These findings suggest that CabZIP2 is involved in plant bacterial disease resistance, potentially through the regulation of defense-related gene expression [44].

The MdXEGIP1 gene, isolated from apple (*Malus* × *domestica* Borkh.), was significantly upregulated by MeJA, with greater expression observed in resistant cultivars compared to susceptible ones upon infection by *Botryosphaeria dothidea*. Subsequent analyses revealed that the recombinant MdXEGIP1 protein exhibits substantial inhibitory activity against xyloglucan-specific endo-β-1,4-glucanases (XEGs) from glycoside hydrolase families 12 and 74, and also inhibited the activity of crude XEG enzyme extracts from *B. dothidea*. This suggests that MdXEGIP1 may play a protective role in apple plants against this pathogen by inhibiting XEG activity [45].

In the citrus disease research, γ-aminobutyric acid (GABA) emerged as a promising eco-friendly treatment strategy against Huanglongbing (HLB). GABA application induced a multi-layered defense mechanism by modulating plant hormone levels and influencing the expression of genes involved in biosynthesis and pathogenesis-related (PR) proteins. Elevated GABA levels have been correlated with increased levels of JA, thereby activating JA-mediated pathways [46]. Similarly, postharvest treatment of kiwifruit (*Actinidia deliciosa* cv. *Jinkui*) with MeJA has been shown to significantly mitigate soft rot caused by *Botryosphaeria dothidea*. This treatment enhances the activities of antioxidant and defense-related enzymes, augments the accumulation of total phenolic content, reduces membrane lipid peroxidation, and upregulates the expression of defense-related genes [47].

In blueberry fruit, treatment with MeJA significantly enhanced the activities of antioxidant and defense-related enzymes, thereby inhibiting the progression of gray mold. Research has demonstrated that MeJA treatment increases the activities of phenylalanine ammonia-lyase, cinnamate 4-hydroxylase, and 4-coumarate-CoA ligase in blueberry fruits, resulting in elevated levels of phenolic and flavonoid compounds. Additionally, MeJA treatment induced bursts of nitric oxide (NO) and H_2_O_2_, which function as crucial signaling molecules in plant disease resistance [48].

In banana, transcription factors such as MaNAC5, MaWRKY1, and MaWRKY2 were implicated in the response to *Colletotrichum musae* infection. These transcription factors were upregulated following *C. musae* infection and upon treatment with SA and MeJA. Protein–protein interaction analyses revealed that MaNAC5 physically interacts with MaWRKY1 and MaWRKY2. These transcription factors can individually or cooperatively activate the transcriptional activity of pathogenesis-related genes such as MaPR1-1, MaPR2, MaPR10c, and MaCHIL1, highlighting their role in SA- and MeJA-induced pathogen resistance. Alterations in the expression of these genes can serve as molecular markers for banana fruit resistance to *C. musae* [49].

In strawberry (*Fragaria vesca* L.), the ectopic expression of the *Arabidopsis thaliana NPR1* gene (AtNPR1) has been shown to enhance resistance to anthracnose, powdery mildew, and angular leaf spot. This resistance was positively correlated with the relative expression levels of AtNPR1 in the transgenic plants. Notably, unlike AtNPR1-overexpressing *Arabidopsis* plants, the transgenic strawberry plants exhibited dwarfism, with most unable to produce stolons or fruits. Additionally, these transgenic lines constitutively expressed the defense gene FvPR5, suggesting distinct differences in the mechanisms of systemic acquired resistance activation between strawberry and *Arabidopsis* [50]. Furthermore, the pre-harvest application of MeJA on strawberry (*Fragaria chiloensis*) fruit resulted in the upregulation of defense-related genes, including β-1,3-glucanase, chitinase, and polygalacturonase-inhibiting protein, thereby reducing the incidence of postharvest gray mold caused by *Botrytis cinerea* [51].

In safflower (*Carthamus tinctorius*), treatment with MeJA was correlated with the upregulation of CtCYP82G24 expression. Among various experimental conditions, including light, dark, and polyethylene glycol (PEG) treatments, CtCYP82G24 exhibited the highest expression levels under MeJA treatment. Transgenic plants overexpressing CtCYP82G24 demonstrated elevated expression of other critical genes involved in flavonoid biosynthesis and accumulated greater quantities of flavonoids and anthocyanins compared to wild-type and mutant counterparts. Conversely, silencing of the CtCYP82G24 gene resulted in diminished flavonoid and anthocyanin accumulation and reduced expression of essential flavonoid biosynthesis genes, underscoring the pivotal role of CtCYP82G24 in MeJA-induced flavonoid accumulation in safflower. These findings suggest that modulation of CtCYP82G24 expression should be considered when employing MeJA to enhance resistance in safflower [52]. In *Cymbidium faberi*, MeJA constitutes a significant component of its floral scent, and pathogenesis-related homologous proteins may be involved in the MeJA signaling pathway [53].

In *Salvia miltiorrhiza*, a comprehensive genome-wide analysis of the auxin response factor (ARF) gene family has identified 25 SmARF gene family members exhibiting distinct expression patterns across various organs, root tissues, and in response to MeJA or indole-3-acetic acid (IAA) treatments. The analysis predicts that SmARF25, SmARF7, SmARF16, and SmARF20 are implicated in the development of flowers, leaves, stems, and roots, respectively. This suggests that MeJA treatment may regulate the growth, development, and disease resistance of *S. miltiorrhiza* by modulating the expression of these genes [54].

MeJA has been observed to confer tolerance in red pine to *Chrysoporthe austroafricana* by modulating the plant’s hormonal equilibrium [55]. In investigations concerning plant-parasitic nematodes, specifically *Pratylenchus zeae* and *Helicotylenchus* spp., treatments with JA and MeJA have been shown to enhance nematode mortality, thereby demonstrating their nematicidal properties [56]. Additionally, the application of exogenous MeJA can activate terpene-based induced defenses. The large-scale transcriptional responses primarily involve genes associated with terpenoid and phenolic biosynthesis, which adversely impacted the feeding behavior of *Monochamus alternatus* adults. This indicates that the induction of terpene-based defenses in pine trees could be instrumental in controlling the dissemination of pine wilt disease [57]. The transcript level of the pathogenesis-related (PR) gene EgrPR2 was reduced under high-concentration MeJA treatment, while the expression of EgrPR3 and EgrLOX is diminished under high-concentration SA treatment, indicating an antagonistic interaction between SA and MeJA. EgrPR2 may serve as a potential diagnostic marker for SA, whereas EgrPR3, EgrPR4, and EgrLOX could act as indicators for MeJA signaling [58].

**Figure 5:**
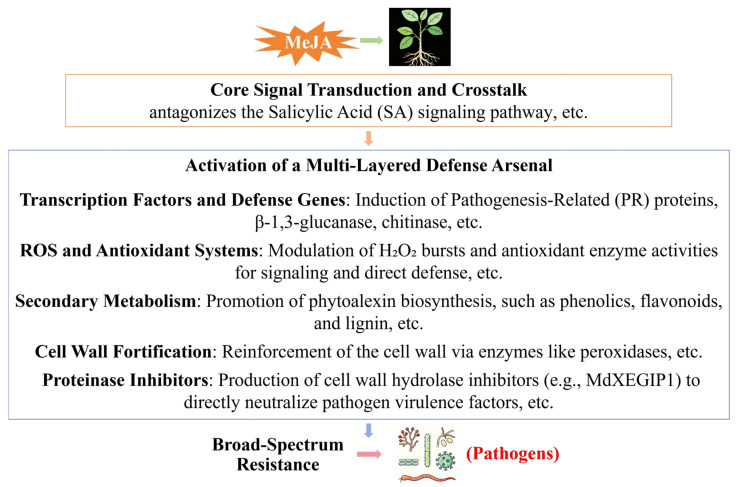
Mechanisms of Methyl Jasmonate (MeJA)-Induced Disease Resistance in Plants [34,35,36,37,39,40,41,42,43,44,45,46,47,48,49,51,52,55,57].

In conclusion, MeJA functions as a crucial plant signaling molecule that systemically activates a plant’s multi-layered defense mechanisms. Its mode of action entails a sophisticated signal transduction network that bolsters plant resistance to pathogens hinges on activating a tailored suite of defenses: antimicrobial proteins in some crops (e.g., apple), antioxidant and phenolic barriers (e.g., kiwifruit, blueberry), specialized metabolite accumulation, and the expression of defense-related genes (Figure 5). Consequently, the exogenous application of MeJA represents promising application prospects in sustainable agriculture. A critical consideration is the timing and method of application to avoid pleiotropic effects on growth and yield, especially in fruiting crops with optimizing elicitation protocols for specific crop-pathogen combinations and integrating MeJA treatments into existing integrated pest management (IPM) frameworks for sustainable horticultural production. The primary goal is to harness this induced resistance strategically without compromising the esthetic and market-quality traits that define horticultural products.

### 2.3. Monitoring the Effects of MeJA Treatment

Accurate detection of the effects of MeJA treatment is essential for evaluating its role in enhancing plant disease resistance. Monitoring the incidence and progression of plant diseases following MeJA application is of significant importance for implementing timely preventive and control measures.

In research investigating the resistance of cacao genotypes to *Ceratocystis cacaofunesta*, liquid chromatography-mass spectrometry metabolomic screening, utilizing high-resolution mass spectrometry, effectively distinguished between resistant and susceptible genotypes. The study identifies that various metabolites, such as linolenic acid, linoleic acid, oleic acid, methyl jasmonate, jasmonic acid, and hydroxylated jasmonates, exhibited specific distribution patterns and content variations in resistant genotypes. These metabolite characteristics can serve as biomarkers for detecting the effects of MeJA treatment and for assessing the resistance of cacao genotypes to *C. cacaofunesta* [59].

In tea plants, the application of exogenous MeJA has been shown to upregulate the transcript levels of the CsOCS gene, consequently enhanced the emission of ocimene in tea leaves. The CsOCS gene encodes an ocimene synthase enzyme responsible for catalyzing the production of (E)-β-ocimene and (Z)-β-ocimene. Variations in the expression of this gene and the increased emission of ocimene may serve as valuable indicators for assessing the impact of MeJA treatment on the defense responses of tea plants. This, in turn, contributes to a more comprehensive understanding of the mechanism of action of MeJA in tea plants under stress conditions [60].

The response of grapevines to *Plasmopara viticola* infection was closely linked to the accumulation of phytoalexins. Grape varieties with varying levels of resistance exhibit differential induction of phytoalexins following infection. The immune-type grape variety, *Muscadinia rotundifolia* ‘Noble’, demonstrated a more rapid and elevated accumulation of phytoalexins, such as resveratrol, upon inoculation with *P. viticola*. This was accompanied by some accumulation of pterostilbene and ε-viniferin. In contrast, the susceptible variety, *Vitis vinifera* ‘Thompson Seedless’, exhibited lower levels of phytoalexin accumulation. Additionally, the concentrations of SA, MeJA, and abscisic acid (ABA) were consistently higher in ‘Noble’ compared to ‘Thompson Seedless’ post-inoculation. Exogenous hormone induction experiments further revealed that phytoalexin accumulation is modulated by plant hormones. These alterations in hormone and phytoalexin levels can serve as indicators of the resistance response of grapevines to downy mildew following MeJA treatment, thereby providing a crucial foundation for disease monitoring [61].

Upon infection by *Botrytis cinerea*, tomato plants exhibited alterations in the emission of biogenic volatile organic compounds (BVOCs), predominantly activating the JA signaling pathway, thereby enabling the regulation of defense mechanisms to counteract the fungal invasion. Conversely, infection by the powdery mildew *Oidium neolycopersici* induced only minor modifications in BVOC emissions, accompanied by the additional release of the sesquiterpene α-copaene. Furthermore, in response to an attack by the aphid *Myzus persicae*, changes in BVOC emissions were detectable even prior to the manifestation of visual symptoms, with the plants emitting methyl salicylate, which triggered the SA-mediated defense pathway. Consequently, the monitoring of BVOC emissions serves as an effective approach for the early detection of responses in MeJA-treated tomato plants to various pathogens, offering a valuable method for the early monitoring of tomato diseases [62].

In peach (*Prunus persica* L.) fruit, MeJA mitigates chilling injury by modulating the fatty acid composition of phospholipids and activating the JA-mediated C-repeat binding factor pathway. Treatment with MeJA resulted in the suppression of PpPAL1, PpPPO1, and PpPOD1/2 gene expression. The enrichment of linoleic acid in potential lipid biomarkers, particularly within phosphatidylcholine, phosphatidylethanolamine, and phosphatidylglycerol, was associated with decreased expression of PpFAD8.1 and increased expression of PpLOX3.1, along with elevated JA content. In the JA signaling pathway, MeJA significantly upregulated the expression of PpMYC2.2 and PpCBF3, while downregulating PpMYC2.1 expression. These characteristics may serve as reliable detection indicators [63].

An enzyme-linked immunosorbent assay (ELISA) method utilizing monoclonal antibodies has been developed to enable the simultaneous detection of JA and MeJA in plant samples. This assay employs a JA-bovine serum albumin (BSA) conjugate as the immunogen for the preparation of monoclonal antibodies. The half-maximal inhibitory concentration (IC_50_) for MeJA was determined to be 2.02 ng/mL, with a limit of detection (LOD) of 0.20 ng/mL. Through the integration of two extraction methods with ELISA detection, it was observed that the concentration of JA in plant samples is approximately 3–5 times higher than that of MeJA, and the ELISA results demonstrated a strong correlation with those obtained via high-performance liquid chromatography (HPLC) [64].

In the context of plant-specific defense responses, identifying reliable molecular markers is essential for accurately evaluating MeJA-induced plant resistance. Molecular marker technology offers precise and effective tools for the assessment of disease resistance.

## 3. Application Strategies of MeJA-Induced Plant Resistance

MeJA modulates plant susceptibility to diseases by influencing growth and developmental processes. Within the framework of integrated disease management, MeJA operates through multiple pathways and can synergistically interact with other control strategies to enhance the efficacy of disease prevention and control measures.

### 3.1. Fundamental Mechanisms of MeJA-Induced Effects

MeJA actively regulates plant growth, facilitates the synthesis and accumulation of metabolites, and exhibits diverse effects across various plant species.

In lettuce seedlings, MeJA significantly enhanced root hair formation by modulating the activity of plasma membrane H^+^-ATPase [65]. Hydroponically grown lettuce treated with MeJA exhibited a marked increase in total chlorophyll and phenolic content, along with significant elevations in glucosinolate and total flavonoid levels, as well as improved antioxidant indices [66]. Additionally, research indicates that exogenous MeJA application promoted flavonoid synthesis and accumulation in *Zanthoxylum bungeanum* [67], induced polyphenol accumulation and related gene expression in *Salvia viridis* hairy roots [68], enhanced selaginellin accumulation in *Selaginella convoluta* [69], triggered oxidative/nitrosative stress and the accumulation of antioxidant metabolites in *Phoenix dactylifera* L. [70], increased glucosinolate biosynthesis, sulforaphane accumulation, and antioxidant activity in broccoli [71], improved lutein and glucosinolate accumulation in kale sprouts [72], augmented drought resistance in grapevines [73], leaded to increases in dry matter, chlorophyll, and carotenoid content, as well as elevated levels of soluble protein, flavonoids, lignin, and enzyme activity in *Isatis indigotica* Fort., conferring a degree of stress resistance [74].

In callus cultures, treatment with MeJA has been shown to induce the accumulation of salidroside, rosavin, and flavonoids, as well as the expression of associated biosynthesis genes in *Rhodiola imbricata* (Edgew.). This treatment also enhances polysaccharide content and enzyme activity, thereby facilitating biomass accumulation and the production of phenolic compounds [75,76]. Furthermore, MeJA promoted tanshinone accumulation in the solid callus of *Salvia miltiorrhiza* [77], increased antioxidant enzyme activity and artemisinin content in in vitro callus of *Artemisia maritima* L. [78], and elevated levels of quinic acid, kaempferol, and glucose in *Damnacanthus major* calli [79].

Additionally, pre-harvest application of MeJA to tobacco leaves significantly enhanced the accumulation of recombinant proteins, which can be leveraged for biopharmaceutical production in plants [80]. In *Kalanchoë blossfeldiana*, MeJA treatment markedly altered the conventional accumulation patterns of anthocyanins in leaves and stems [81]. Moreover, MeJA plays a crucial role in mitigating cadmium (Cd) stress; exogenous application of MeJA reduced cadmium uptake by plant roots while increasing cadmium sequestration in the root cell wall, thereby hindering the root-to-shoot translocation of Cd within the plant [82,83].

MeJA demonstrates diverse effects in the context of fruit storage. The pre-harvest application of MeJA on strawberries has been shown to preserve fruit firmness and anthocyanin content while delaying the onset of decay [84]. In postharvest scenarios, MeJA treatment has been observed to mitigate fruit weight loss, enhance catalase and peroxidase activities, and augment the overall antioxidant capacity of the fruit [85]. Specifically, the pre-harvest application of MeJA on blackcurrant (*Ribes nigrum* L.) has significantly increased anthocyanin and flavonol content, concurrently enhanced the antioxidant properties of the fruit [86]. In the case of pomegranate (*Punica granatum*), MeJA treatment was effective in maintaining fruit quality by reducing weight loss, peel hardening, and browning, as well as decreasing the incidence of chilling injury [87]. Furthermore, postharvest MeJA treatment of olive (*Olea europaea*) fruit has been successful in prevented alterations in fatty acid composition and the loss of phenolic acids during storage [88]. In papaya (*Carica papaya*), postharvest MeJA treatment has significantly reduced fruit weight loss, disease incidence, and chilling injury index [89]. Postharvest application of jasmonates to peach fruit has been shown to mitigate internal flesh browning without altering phenolic content, while also decreasing relative electrolyte leakage, which suggests reduced membrane damage and enhanced chilling tolerance [63,90].

Evidently, MeJA can extensively regulate plant physiology and secondary metabolism. Central to this regulatory function is MeJA’s role as a pivotal signaling molecule. The activation of plant defense networks facilitated by MeJA is crucial not only for resistance to abiotic stresses but also for effective responses to biotic stresses, including pathogen infections.

### 3.2. Combined Application of MeJA and Biological Control

The integration of MeJA with biological control methods can significantly enhance the efficacy of disease management, offering a sustainable and environmentally benign approach to plant disease control.

A study investigating the interaction between wheat (*Triticum aestivum* L.) and the spot blotch pathogen (*Bipolaris sorokiniana*) demonstrated that MeJA concentrations exceeding 100 mg/L effectively inhibited the spore germination of *B. sorokiniana* under laboratory conditions. The concurrent application of MeJA at 150 mg/L and the biological control agent *Trichoderma harzianum* UBSTH-501 was found to augment indole acetic acid production in the wheat rhizosphere, thereby facilitating plant growth and development. Furthermore, this combination enhanced the activities of defense-related enzymes, including catalase, ascorbate peroxidase, phenylalanine ammonia-lyase, and peroxidase, and increased the accumulation of SA, as well as total free phenolics, ferulic acid, caffeic acid, coumaric acid, and chlorogenic acid. The synergistic interaction between exogenous MeJA and the biocontrol agent significantly mitigated disease severity and progression, suggesting that this combination induces JA- and/or SA-dependent defense signaling pathways. Consequently, it enhances wheat resistance to spot blotch by stimulating enzyme activities and phenolic compound accumulation [91].

A field study on cotton demonstrated that the application of exogenous MeJA enhanced the emission of cotton volatiles and the secretion of nectar, while not influencing cotton yield. Although MeJA treatment modified the induced responses of cotton plants, it exerted minimal effects on the insect populations present on the plants. This study suggests a preliminary avenue for employing MeJA in conjunction with biological control methods to modulate plant-insect interactions. Further investigation is required to optimize this approach for improved pest management [92].

In the management of bacterial wilt caused by *Ralstonia solanacearum* in tomato (*Solanum lycopersicum*), the *Bacillus amyloliquefaciens* strain WF02 demonstrated significant efficacy. The research revealed that treatment with this strain led to the upregulation of genes associated with the salicylic acid pathway, specifically phenylalanine ammonia-lyase and pathogenesis-related proteins. In contrast, the gene related to the jasmonic acid pathway, lipoxygenase, was upregulated exclusively in the susceptible cultivar L390. These findings suggest that strain WF02 can confer protection against bacterial wilt in susceptible tomato cultivars through both direct and indirect mechanisms, involving the jasmonic acid regulatory pathway [93]. Additionally, a study investigating the application of *Bacillus thuringiensis* var. *kurstaki* (Bt) for controlling corn earworm (*Helicoverpa zea*) in tomatoes found that treating tomato plants, which had been subjected to feeding by *H. zea* or induced by jasmonate, with a commercial Bt formulation resulted in increased mortality of *H. zea*. The elevated mortality rate of *H. zea* larvae was strongly correlated with increased polyphenol oxidase (PPO) activity in leaf tissues, while peroxidase (POD) activity showed only a weak correlation with Bt-induced mortality, indicating that plant-induced defense responses can act synergistically with microbial insecticides [94].

In research focused on the management of Verticillium wilt (*Verticillium dahliae*) in olive trees (*Olea europaea*), the application of *Aureobasidium pullulans* AP08, *Bacillus amyloliquefaciens* PAB-024, and copper phosphite (CuPh), either individually or in combination, was found to significantly reduce the disease incidence. This reduction was attributed to the enhancement of plant defense-related enzyme activity and the modulation of hormone levels, among other mechanisms. Notably, *A. pullulans* and CuPh, when applied either to the foliage or the roots, demonstrated the most pronounced effects [95].

### 3.3. Combination of MeJA with Other Control Pathways

In watermelon, research has demonstrated that the application of exogenous melatonin and MeJA could enhance resistance to *Fusarium oxysporum* f. sp. *niveum* race 2 (FON2) in a dose-dependent manner. Exogenous melatonin significantly promoted the upregulation of genes involved in MeJA synthesis and elevated MeJA levels following FON2 infection. Conversely, pretreatment with a MeJA synthesis inhibitor attenuated the melatonin-induced resistance to FON2. Additionally, MeJA facilitated the upregulation of the melatonin biosynthesis gene caffeic acid O-methyltransferase 1 (ClCOMT1) and enhanced melatonin accumulation during FON2 infection. The diminished resistance to FON2 resulting from the deletion of ClCOMT1 can be completely restored by the application of exogenous MeJA. These findings suggest that melatonin and MeJA establish a mutually reinforcing positive regulatory loop in response to FON2 infection. Furthermore, polyphenol oxidase, phenylalanine ammonia-lyase, and lignin were also implicated in the MeJA-induced resistance mechanism against FON2 [96].

In peanut (*Arachis hypogaea*) hairy root cultures, the combined application of MeJA and methyl-β-cyclodextrin (CD) facilitated the sustained accumulation of high concentrations of resveratrol, piccatannol, arachidin-1, and arachidin-3 in the culture medium, with average yields reaching specific quantitative values. This synergistic interaction, as opposed to the application of MeJA or CD individually, significantly enhanced the expression of the resveratrol synthase gene, thereby augmenting resveratrol synthesis. These findings suggest that peanut hairy root cultures can function as a controllable and sustainable sterile system for the production of various bioactive stilbenoids. Furthermore, this study highlights the potential of innovative combination treatments to stimulate the accumulation of plant secondary metabolites [97].

In ‘Hass’ avocado (*Persea americana* ‘Hass’) fruit, vapor treatment with MeJA and methyl salicylate (MeSA) significantly reduced the incidence of anthracnose caused by *Colletotrichum gloeosporioides* Penz. This reduction was attributed to the enhancement of defense-related enzyme activities, including chitinase, β-1,3-glucanase, and phenylalanine ammonia-lyase, as well as an increase in epicatechin content [98].

The synergistic interaction between MeJA and other pathways presents a novel approach for augmenting plant resistance to pathogens, thereby offering a promising strategy for plant disease management. In practical applications, it is crucial to carefully consider the concentration and timing of MeJA application, along with its synergistic interactions with other control measures, to achieve optimal disease prevention and control outcomes. Consequently, further investigation into the mechanisms of action and application strategies of MeJA across various crops and disease systems, as well as the optimization of its combinations with other control methods, is essential to provide more effective support for sustainable agricultural development.

## 4. Challenges in MeJA-Induced Resistance

### 4.1. Safety and Practical Application Considerations

Although MeJA demonstrates considerable efficacy in enhancing plant disease resistance, its safety and environmental implications necessitate more comprehensive investigation. In terms of plant growth and development, studies have indicated that MeJA exerts differential effects across various plant species, with elevated concentrations potentially inhibiting growth. For instance, in pea plants, pretreatment with relatively high concentrations of MeJA was observed to impair growth characteristics, decrease photosynthetic rate and stomatal conductance, induce stomatal closure, and increase leaf cell mortality [99].

From an environmental standpoint, the widespread application of MeJA may exert significant impacts on ecosystems. MeJA has the potential to affect non-target organisms. For example, MeJA treatment can modify the emission of plant volatiles, which may, in turn, influence the behavior and ecology of surrounding insects [100]. This alteration could attract or repel beneficial insects, thereby indirectly disrupting ecological balance [101,102]. Furthermore, there is a notable deficiency in research concerning the persistence, degradation pathways, and potential metabolites of MeJA in environmental contexts. The long-term accumulation of MeJA and its effects on soil microbial communities, aquatic ecosystems, and other environmental components remain uncertain. Consequently, it is imperative to conduct comprehensive assessments of MeJA’s potential environmental impacts and its effects on non-target organisms to ensure the safety and sustainability of its application.

To effectively translate the promising efficacy of MeJA from controlled experiments to widespread agricultural practice, several critical issues regarding cost, scalability, and sustainability must be addressed. Firstly, a rigorous cost–benefit analysis is essential. The relatively high cost of synthetic MeJA and its application may currently limit its use to high-value crops unless the economic returns from improved yield, quality (e.g., enhanced secondary metabolite content), and reduced pesticide use are conclusively demonstrated to outweigh the inputs [103]. Secondly, challenges in large-scale application persist, including the optimization of efficient and stable delivery formulations (e.g., controlled-release systems) to counteract its volatility, the standardization of application protocols for different crops, and the need for environmental fate studies to ensure regulatory compliance [104]. Finally, exploring cheaper and more environmentally friendly alternatives is a crucial research direction. This includes the investigation of other natural or synthetic elicitors (e.g., chitosan, oligosaccharides), plant-derived extracts, and breeding strategies for genotypes with constitutively enhanced defense pathways, which could offer more sustainable crop protection solutions [6].

### 4.2. Durability and Stability of MeJA-Induced Resistance

The durability and stability of MeJA-induced plant resistance is a pivotal concern, as it directly influences its practical effectiveness in disease management. The impact of MeJA induction is modulated by various factors. For instance, in the interaction between rice and the blast fungus *Magnaporthe oryzae*, temperature plays a significant role in modulating MeJA-mediated resistance. Research indicates that at the optimal rice growth temperature of 28 °C, *M. oryzae* effectively triggered the expression of JA biosynthesis and signaling genes in rice. In contrast, at 22 °C, this induction was not observed, resulting in a more severe incidence of rice blast. The application of MeJA emerges as an effective strategy to enhance resistance against rice blast in warmer climates, thereby providing a crucial theoretical foundation for managing rice blast in the context of climate change [105].

Several studies have demonstrated that MeJA-induced resistance can effectively protect plants from pathogen infection for a limited duration, although this resistance may diminish over time. In an experiment involving 2-year-old spruce seedlings treated with MeJA and subsequently inoculated with the necrotrophic fungus *Grosmannia penicillata* (transmitted by *Ips typographus*), MeJA treatment was observed to reduce the extent of necrotic lesions in the bark after 8 weeks, thereby indicating the induction of long-term resistance. However, transcriptional analysis revealed that the upregulation of genes involved in jasmonate, salicylate, and ET biosynthesis, as well as downstream signaling pathways induced by MeJA treatment, was transient. This suggests a potential weakening of the associated defense responses over time [106]. In contrast, the resistance of wheat high-temperature seedling-plant (HTSP) to stripe rust (*Puccinia striiformis* f. sp. *tritici*, Pst) is characterized as non-race-specific and durable, yet the role of MeJA in this process is complex. Research has identified that the TaWRKY70 gene plays a positive role in HTSP resistance, with its expression being influenced by various factors, including temperature and hormonal signals. Notably, MeJA treatment was found to reduce TaWRKY70 expression, which might affect defense responses dependent on this gene and consequently impact the durability of resistance [107]. In grape (*Vitis vinifera*) cell cultures, encapsulation of MeJA in poly(lactic-co-glycolic acid) (PLGA) nanoparticles facilitated more rapid cellular uptake and activation of MeJA-induced responses compared to free MeJA, thereby demonstrating an enhanced biological response. However, practical applications may be influenced by factors such as nanoparticle stability, release characteristics, and variability in effects across different grape varieties and growth conditions, which could impact the consistency of MeJA-induced resistance [108]. In tea plants (*Camellia sinensis*), exogenous application of MeJA effectively enhanced reactive oxygen species (ROS) scavenging under cold stress, thereby maintaining cell membrane stability. The R2R3-MYB transcription factor genes were significantly upregulated under the combined treatment of MeJA and cold stress, indicating their involvement in both the cold stress signal response and the JA signaling pathway. Nonetheless, the stability of gene expression across various tea plant varieties and developmental stages, as well as the long-term effects on the efficacy of MeJA-induced resistance, warrant further investigation [109].

Consequently, it is imperative to acquire a comprehensive understanding of the mechanisms underpinning the durability and stability of MeJA-induced resistance, as well as to identify strategies for sustaining or extending this resistance, in order to fully harness the potential of MeJA in disease management. Future research should prioritize elucidating the molecular regulatory network associated with MeJA-induced resistance and investigate approaches to enhance the durability and stability of this resistance through the modulation of relevant gene expression or signaling pathways.

## 5. Challenges and Future Perspectives

It is anticipated that the potential of MeJA in disease management will be further elucidated, thereby offering robust support for the advancement of sustainable agricultural development.

To unlock the full potential of MeJA as a sustainable crop protection tool, future research should prioritize addressing the following five key questions: How can we precisely modulate the crosstalk between MeJA and other hormone signaling pathways (e.g., SA, ET) to optimize the growth-defense balance in different crops and under varying stress scenarios? What is the role of epigenetic mechanisms (e.g., DNA methylation, histone modification) in establishing, maintaining, and transmitting MeJA-induced resistance, and can they be harnessed to create durable “primed” states? How can we design precise and efficient MeJA delivery systems (e.g., nano-formulations, controlled-release technologies) tailored to specific crop-pathogen systems and growth stages to maximize efficacy and minimize environmental impact? What are the long-term ecological consequences of field-scale MeJA application, particularly its effects on non-target organisms (beneficial insects, soil microbiota) and ecosystem functions? How can MeJA be optimally integrated with other disease management strategies (resistant cultivars, biocontrol agents, cultural practices) within a robust Integrated Pest Management (IPM) framework to achieve synergistic and sustainable control?

## Data Availability

No new data were created or analyzed in this study. Data sharing is not applicable to this article.

## Abbreviations

The following abbreviations are used in this manuscript:

MeJA	Methyl jasmonate
JA	jasmonic acid
FNSI	Flavone synthase I
cAMP	cyclic adenosine monophosphate
FIN219	FAR-RED INSENSITIVE 219
ABA	abscisic acid
FHB	Fusarium head blight
DON	deoxynivalenol
PKA	protein kinase A
BBTI	Bowman-Birk type protease inhibitor
Abm	monooxygenase
12OH-JA	12-hydroxy-JA
Z-3-HAC	Z-3-hexenyl acetate
PR1	pathogenesis-related protein 1
TRXM	thioredoxin M-type chloroplast precursor
ROS	reactive oxygen species
MYMIV	Mungbean Yellow Mosaic India Virus
H_2_O_2_	hydrogen peroxide
PAL5	phenylalanine ammonia-lyase
BSMT	benzoic acid/salicylic acid carboxyl methyltransferase
ICS	isochorismate synthase
OA	oxalic acid
XEGs	xyloglucan-specific endo-β-1,4-glucanases
GABA	γ-aminobutyric acid
HLB	Huanglongbing
NO	nitric oxide
AtNPR1	*Arabidopsis thaliana NPR1* gene
ARF	auxin response factor
IAA	indole-3-acetic acid
BVOCs	biogenic volatile organic compounds
ELISA	enzyme-linked immunosorbent assay
MeSA	methyl salicylate
CD	methyl-β-cyclodextrin
FON2	*Fusarium oxysporum* f. sp. *niveum* race 2
ClCOMT1	caffeic acid O-methyltransferase 1
CuPh	copper phosphite
